# MiRNAs and Their Role in Venous Thromboembolic Complications

**DOI:** 10.3390/diagnostics13213383

**Published:** 2023-11-04

**Authors:** Ilgiz Gareev, Valentin Pavlov, Weijie Du, Baofeng Yang

**Affiliations:** 1 Central Research Laboratory, Bashkir State Medical University, 3 Lenin Street, 450008 Ufa, Russia; ilgiz_gareev@mail.ru; 2 Department of Urology, Bashkir State Medical University, 3 Lenin Street, 450008 Ufa, Russia; pavlov@bashgmu.ru; 3 Department of Pharmacology, The Key Laboratory of Cardiovascular Research, Ministry of Education, College of Pharmacy, Harbin Medical University, Harbin 150067, China; 4 Translational Medicine Research and Cooperation Center of Northern China, Heilongjiang Academy of Medical Sciences, Harbin 150081, China

**Keywords:** miRNA, venous thromboembolic complications, cardiovascular disease, hemostasis, therapeutic targets, biomarkers

## Abstract

Venous thromboembolic complications (VTCs), which include deep vein thrombosis (DVT) and pulmonary embolism (PE), have remained a pressing problem in modern clinical medicine for a long time. Despite the already wide arsenal of modern methods for diagnosing and treating this disease, VTCs rank third in the structure of causes of death among all cardiovascular diseases, behind myocardial infarction (MI) and ischemic stroke (IS). Numerous studies have confirmed the importance of understanding the molecular processes of VTCs for effective therapy and diagnosis. Significant progress has been made in VTC research in recent years, where the relative contribution of microRNAs (miRNAs) in the mechanism of thrombus formation and their consideration as therapeutic targets have been well studied. In this case, accurate, timely, and as early as possible diagnosis of VTCs is of particular importance, which will help improve both short-term and long-term prognoses of patients. This case accounts for the already well-studied circulating miRNAs as non-invasive biomarkers. This study presents currently available literature data on the role of miRNAs in VTCs, revealing their potential as therapeutic targets and diagnostic and prognostic tools for this terrible disease.

## 1. Introduction

Venous thromboembolic complications (VTCs), which include deep vein thrombosis (DVT) and pulmonary embolism (PE), remain the most important problem in clinical medicine and affect the professional sphere of doctors of all specialties without exception. The importance of VTCs is due to their extremely high potential risk to the health and life of the patient. Improving the quality of prevention, diagnosis, prognosis, and treatment of VTCs can save the lives of thousands of people and ensure a noticeable reduction in financial pressure on the healthcare budget, thanks to the prevention of severe disabling diseases [1,2,3].

Currently, there are effective surgical and drug treatments for VTCs that can reduce disability in the population. However, the early diagnosis and prognosis of the disease is a key factor in the success of the treatment and is directly related to understanding the pathogenesis of VTCs, which leads to the possibility for forming risk groups for PE among patients with DVT [4]. Currently, the mechanisms of the incomplete resolution of venous thrombi in patients with DVP and patients who have suffered PE are completely unknown. It has been shown that the process of the fibrotic transformation of thrombotic masses is inextricably linked with the activities of inflammation and angiogenesis, as well as changes in the hemostasis system, in the regulation of which microRNAs (miRNAs) may take part [5,6]. It has been proven that miRNAs play a significant role in various biological processes, including the cell cycle, apoptosis, proliferation, and differentiation, regulating the expression of about 90% of all human genes. In addition, their direct role in the pathogenesis of many human diseases, including the imbalance of the hemostatic system, such as thrombosis, has already been proven (Table 1) [7,8,9,10,11,12,13,14,15,16].

**Figure 1:**
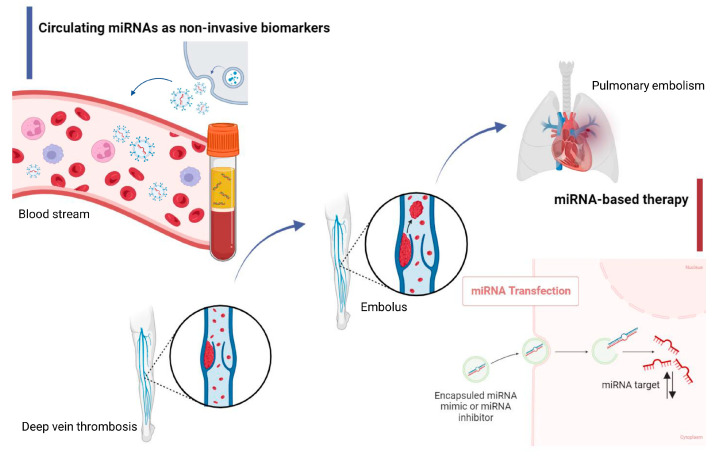
Schematic illustration of the capabilities of microRNAs (miRNAs) in venous thromboembolic complications (VTCs). There are two directions for studying miRNAs in deep vein thrombosis (DVT) and pulmonary embolism (PE): (1) the transfection of a miRNA mimic or inhibitor to change the expression of a target miRNA with the possibility of therapeutic interventions and (2) the consideration of circulating miRNAs as non-invasive biomarkers for the purpose of diagnosing DVT and the possibility of prognosticating PE.

The relevance of the problem of PE is due to the difficulties of timely diagnosis due to the polymorphism of clinical syndromes. To detect PE, various research methods are used—both laboratory and instrumental. The degree of their information content varies [17]. Among the most important, from the point of view of PE verification, are methods, such as the determination of the D-dimer content, electrocardiography, ultrasound (echocardiography), and radiation methods (chest radiography, lung ventilation perfusion scan (VQ Scan), computed tomography (CT) of the chest, and angiopulmonography) [18]. The determination of the D-dimer content, ultrasonography, and venography are most often used in the diagnosis of DVT, after surgery [19]. Ultrasonography has certain limitations. In contrast, venography is invasive and increases the risk of complications, including blood clot rupture and allergic reactions, which also limit its use [19]. One solution to this problem is to find accurate, effective, and non-invasive methods for diagnosing VTCs. And in this regard, circulating miRNAs are already being studied as potential biomarkers in VTCs. Circulating miRNAs can be secreted from cells into human biological fluids as a part of extracellular vesicles (EVs) (microvesicles and exosomes) or can be a part of RNA-protein complexes, such as miRNA/Argonaute-2 (Ago2) and miRNA/nucleophosmin 1 (NPM1) [6,20]. Such miRNAs are resistant to nucleases, are specific to certain pathological conditions, and are easy to detect using modern laboratory methods, which makes them potential biomarkers for patients with VTCs [6]. The discovery of aberrant miRNA expression in VTCs suggests the possibility of creating new diagnostic and prognostic tools. The analysis of miRNA target genes could lead to new horizons in understanding the pathogenesis of VTCs and, thereby, new treatments (Figure 1). In this study, we review preclinical and clinical studies that have examined miRNAs and their target genes in VTCs with the goal of better understanding the course and progression of VTCs and the potential application of miRNAs in clinical practice.

## 2. MiRNAs and Hemostasis

**Figure 2:**
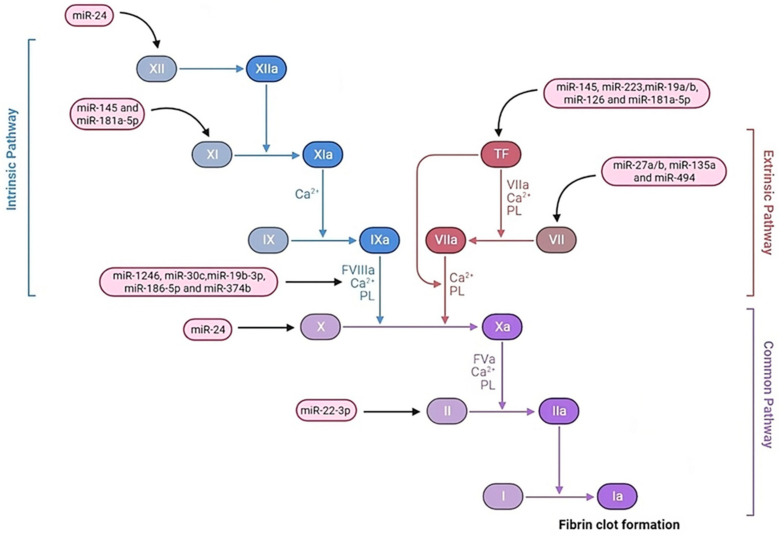
Schematic illustration of microRNA (miRNA) regulation of blood coagulation and fibrinolysis pathways. Several miRNAs are presented that are reported to regulate key factors of hemostasis. Note: TF, Tissue factor; PL, Platelets.

**Figure 3:**
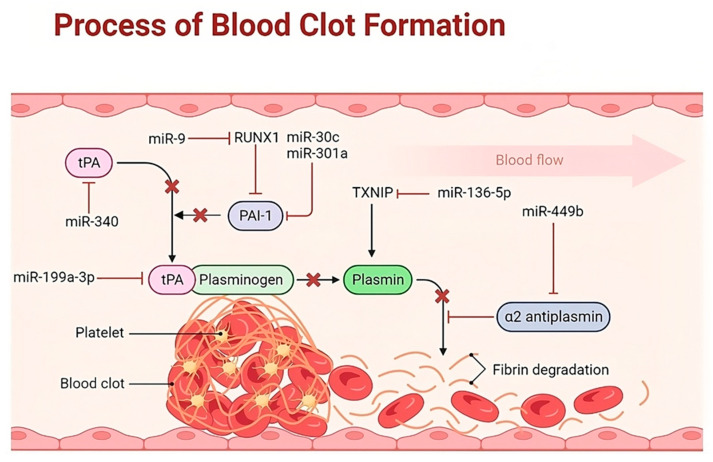
The illustration of a panel of regulatory miRNAs potentially involved in the process of blood clot formation. The normal coagulation pathway is a balance between the procoagulant pathway responsible for thrombus formation and the mechanisms that inhibit it beyond the site of injury. This delicate balance can be disrupted whenever the procoagulant activity of coagulation factors increases or the activity of natural inhibitors decreases under the regulatory action of microRNAs (miRNAs). Note: tPA, Tissue plasminogen activator; PAI-1, Plasminogen activator inhibitor-1; RUNX1, Runt-related transcription factor 1; TXNIP, Thioredoxin interacting protein.

**Figure 4:**
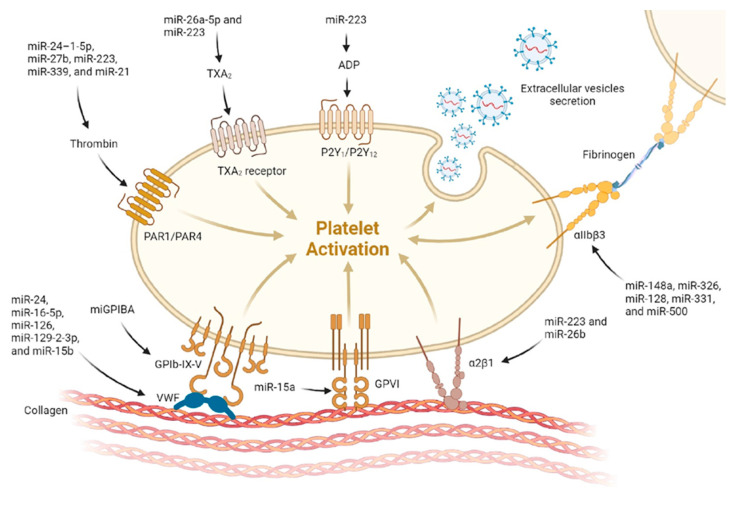
MicroRNA (miRNA) regulation of platelet activity. Abundant evidence demonstrates a critical role for miRNAs in platelet biology and platelet production and activation. Moreover, from the literature, activated platelets release extracellular vesicles (EVs) (exosomes and microvesicles), which contain a wide range of proteins and nucleic acids, including miRNAs, such as miR-223, miR-25-3p, miR-339, miR-21, and miR-328.

According to the literature, miRNAs control the expression of several key factors of hemostasis (platelet biogenesis and function, coagulation factors, anticoagulant mechanisms, and fibrinolysis), indicating that the dysregulation of these miRNAs can lead to hemostatic imbalance and, accordingly, increased thrombosis or bleeding (Figure 2, Figure 3 and Figure 4) [21,22,23,24,25,26].

Tissue factor (TF) is a critical factor in the initiation of coagulation, and its overexpression has been extensively studied in patients with cancer complicated by coagulopathy [27]. While the regulatory role of miRNAs was actively being studied for TF, some miRNAs were found to directly inhibit TF expression and were active in various tumor cell lines. For instance, in breast cancer (BC) cell lines, miR-19 directly inhibits TF expression, and miR-19a inhibits TF expression in colon cancer (CC) cells [28,29]. In addition, Li et al. found that the activation of miR-223 resulted in decreased TF expression and the inhibition of tumor necrosis factor-α (TNF-α) expression in the aorta tissue of C57BL/6J mice and cultured endothelial cells (ECs) (EA.hy926 cells and human umbilical vein endothelial cells (HUVECs)) [30]. This study demonstrates the miR-223-mediated suppression of TF expression, suggesting a novel molecular mechanism for controlling the coagulation cascade and suggesting a clue against thrombogenesis during the process of atherosclerotic plaque rupture. The relationships between miR-93, miR-106, and TF were observed in leiomyoma cells, where the suppression of miR-93 and miR-106 expression levels and the associated increase in TF expression promoted inflammatory and metabolic processes in tumor cells [31]. In human microvascular ECs, miR-19a and miR-126 have been shown to modulate endothelial thrombogenicity through the direct inhibition of TF expression and activity. In addition, changes in the expression levels of miR-19a and miR-126 were induced by the inflammatory stimulus, TNF-α.

Teruel et al. demonstrated significantly lower expression levels of miR-19b and miR-20a in monocytes from patients with antiphospholipid syndrome (APS) and systemic lupus erythematosus (SLE) compared with controls, and miR-19b and miR-20a expressions were inversely correlated with TF expression in the corresponding monocytes [32]. Thus, these results showed the role of miR-19b and miR-20a in hypercoagulability in conditions observed in patients with APS and SLE. Also, miRNA has been identified as being associated with plasminogen activator inhibitor-1 (PAI-1), a key modulator of the fibrinolytic pathway. Recently, increased PAI-1 expression and thrombus formation have been associated with decreased miR-30c expression in patients with type 2 diabetes mellitus (DM) [33]. Fibrin formation may be under the control of miR-409-3p, which was identified in HuH-7 cells through miRNA library screening and decreased steady-state levels of all the fibrinogen genes (fibrinogen alpha (FGA), fibrinogen beta (FGB), and fibrinogen gamma (FGG)) [34]. It was found that when miR-409-3p is activated, fibrinogen formation in HuH-7 cells decreases. These data suggest that thrombus growth and persistence may be subject to miRNA-mediated regulation of fibrinolysis through PAI-1 and fibrinogen synthesis.

## 3. MiRNA and DVT

Currently, the molecular mechanisms of DVT pathogenesis are not fully understood, which significantly limits the creation of new therapeutic, diagnostic, and prognostic tools. It is known that the processes of thrombus lysis and the restoration of the venous wall are associated with proinflammatory cytokines, chemokines, and leukocytes. Additionally, the definition of Virchow’s triad considers three major factors that contribute to the formation of blood clots, including changes in blood flow, endothelial damage, and a hypercoagulable state. However, the molecular mechanism of DVT is not fully understood. Recent studies have suggested that miRNAs are involved in the formation and development of DVT. In their study, Zhang et al. observed increased interleukin 6 (IL-6) expression in the peripheral blood mononuclear cells (PBMCs) of patients with DVT and investigated the expression profile of miR-338-5p in patients with DVT, in the HUVEC cell line, and in DVT animal models, using microarrays [35]. The authors provided evidence that miR-338-5p plays an important role as a suppressor of IL-6 by showing that decreased miR-338-5p expression increased IL-6 expression and promoted DVT formation. Tang et al. designed a study for the purpose of verifying the effects of miR-495 targeting interleukin 1 receptor type 1 (IL1R1) on lower extremity DVT through the toll-like receptor 4 (TLR4) signaling pathway in vitro and in vivo. As revealed throughout their study, the overexpression of miR-495 promoted the proliferation and inhibition of the apoptosis of femoral vein ECs through the direct regulation of the 3′ untranslated region (3′-UTR) of messenger RNA (mRNA) IL1R1 expression via the TLR4-signaling pathway [36].

Knowledge accumulated in recent years suggests that DVT is closely associated with the fibrotic remodeling of vein walls, which consists of a switch in the vascular smooth muscle cell (VSMC) phenotype from contractile to synthetic, with VSMC proliferation, collagen deposition, and damage to the extracellular matrix (ECM) by matrix metalloproteinases (MMPs). More recently, the involvement of MMPs together with the tissue inhibitors of metalloproteinases (TIMPs) in the mechanism of DVT has been recognized as significant and is actively being discussed [37,38]. Francis et al. reported that high plasma levels of MMPs, including MMP-1, -2, -3, -7, -8, and -9, are observed in patients with acute DVT [39]. Ai et al. observed a decrease in the expression of miR-411, while the expressions of hypoxia-inducible factor-1α (HIF-1α), Collagen I, as well as MMP-2 were increased in the vein walls and corresponding VSMCs obtained from rats with DVT [40]. HIF-1α is known to play important roles in cell proliferation, migration, and differentiation; vasoconstriction or vasodilation; ECM degradation; and angiogenesis [41,42]. In addition, HIF-1α regulates many genes, and mediates vein wall fibrosis remodeling, implying HIF-1α might be a potential target for the treatment of vein wall fibrosis in DVT [43]. Eventually, the increased expression levels of miR-411 inhibited HIF-1α expression, leading to the downregulation of MMP-2 in VSMCs and, consequently, alleviated vein wall fibrosis in vitro.

Endothelial nitric oxide (NO) regulates several physiological processes, such as platelet adhesion and aggregation, which may influence susceptibility or resistance to thrombosis [44]. There is evidence that the expression level of endothelial nitric oxide synthase (NOS3), which plays an important role in NO synthesis, was significantly reduced in MVEC cell lines by the overexpression of miR-195 and miR-582 [45]. The inhibition of the expression of miR-195 and miR-582 in ECs could promote an increase in NOS3 expression, indicating that NOS3 mRNA was a target for miR-195 and miR-582. In other words, the aberrant expression of miR-195 and miR-582 can cause endothelial dysfunction by regulating vascular homeostasis and can ultimately lead to thrombus formation in both arteries and veins. Table 2 summarizes the results of other studies that assessed the role of some representative miRNAs in DVT pathogenesis [46,47,48,49,50,51,52,53,54,55,56,57,58,59,60,61].

## 4. MiRNA and PE

PE is a critical and potentially life-threatening medical condition characterized by the formation of blood clots, typically in the deep veins of the legs, which can dislodge and travel to the pulmonary arteries, causing obstruction and compromising cardiopulmonary function. Pulmonary hypertension, often resulting from recurrent PE, can lead to progressive respiratory dysfunction and long-term disability. Considering these serious consequences, there is a pressing need for improved innovative therapeutic strategies for PE [62]. Recent advancements in medical research have uncovered a burgeoning interest in the role of miRNAs in the context of PE pathogenesis. Zhu et al. identified miRNA profiles in both in vitro and in vivo models of acute PE [63]. The analysis identified specific miRNAs affected by hypoxia/reoxygenation (H/R) or acute PE induction. Notably, some miRNAs were upregulated (miR-34a-5p, miR-324-5p, and miR-331-3p), while others were downregulated (miR-429, miR-491-5p, and miR-449a). Treatment with urokinase-type plasminogen activator (uPA) effectively restored these miRNA levels to those observed in healthy controls, both in laboratory cell models and in live mice. Additionally, the study involved predicting the target genes of these miRNAs and exploring their potential functions. The identified targets were linked to critical biological processes, including cell growth, proliferation, and inflammation. To further validate their findings, the researchers conducted experiments in which they artificially increased the levels of miR-449a, using a mimic. This manipulation completely reversed the protective effect of uPA in the in vitro H/R model. In acute PE, miR-34a-3p was identified as one of the pivotal miRNAs that directly regulate the expression of dual-specificity phosphatase-1 (DUSP1) [64]. Upregulated miR-34a-3p was found to effectively inhibit the proliferation of pulmonary artery smooth muscle cells (PASMCs), a hallmark of acute PE progression. This suggests that miR-34a-3p may offer potential therapeutic avenues for managing acute PE by targeting pulmonary vascular proliferation.

Chronic thromboembolic pulmonary hypertension (CTEPH) is a rare form of pathology that develops because of the chronic obstruction of large/medium branches of the pulmonary arteries and secondary changes in the microvasculature of the lungs. CTEPH is a late complication of acute PE, with an incidence of 0.1–9.1% during the first two years after the episode [65]. The complex pathogenesis of CTEPH remains not fully understood to date. In an experimental study on a line of PASMCs obtained from the surgical specimens of patients with CTEPH, Wang et al. demonstrated the inhibitory effect of let-7d on cell proliferation by increasing the expression level of the p21 cell-cycle inhibitor mRNA [66]. In other words, the authors concluded that the relationship between the reduced let-7d expression and excessive proliferation of PASMCs in material from patients with CTEPH plays a role in the pathogenesis of the disease. Chen et al., in a study on 190 patients with CTEPH and a control group of patients without CTEPH, confirmed the previously proven association of the development of the disease with the polymorphism of the fibrinogen alpha gene (FGA) and demonstrated the direct role of miR-759 in the regulation of FGA expression through interactions with the polymorphic site (deletion/insertion (Del/Ins)) polymorphism of 28 base pairs (bp) in the 3′-UTRs of mRNAs [67].

Although our comprehension of the role of miRNAs in PE is still in its nascent stages, these findings underscore the potential of miRNAs to be instrumental in unraveling the intricate mechanisms of PE and potentially devising novel treatment strategies. However, it is crucial to emphasize that further research, particularly in larger cohorts and comprehensive confirmatory experiments, is imperative before clinical applications can be fully realized. Table 3 summarizes the results of other studies that assessed the role of some representative miRNAs in PE pathogenesis [68,69,70,71,72,73].

## 5. Circulating miRNAs vs. Traditional Biomarkers

Numerous studies have attempted to find accurate and early diagnostic laboratory biomarkers for PE and DVT. However, to date, plasma D-dimer is the only well-established and clinically applicable biomarker for the identification of PE and DVT. The D-dimer test reflects the level of fibrin degradation. A negative D-dimer result is relative evidence to exclude PE and DVT, but a positive result has low specificity and low diagnostic and prognostic value for VTCs [74]. D-dimer concentrations may also be elevated in non-thrombotic disorders, including disseminated intravascular coagulation (DIC), infection, and stroke [75,76]. In recent years, one of the most promising new biomarkers is soluble P-selectin, which has higher specificity than D-dimer in the non-invasive diagnosis of PE and DVT [77]. However, D-dimer and P-selectin alone or in combination do not have high sensitivity or specificity in diagnosing thrombus formation.

Circulating miRNAs, as biomarkers of PE and DVT, have been less studied, although circulating miRNAs may be more sensitive and accurate in the diagnosis and prognosis of VTCs. Recently, several studies have been carried out showing that circulating microRNAs are preferable as biomarkers because they are highly stable in human biological fluids, such as blood, and can be detected in the early stages of disease development, while protein structures, such as D-dimer, are found in blood only when damage has already occurred [78,79]. In addition, miRNAs play a role in almost all cellular functions, including hemostatic balance [21,22,23,24,25,26].

### 5.1. In DVT

Wang et al. found that among the 13 plasma miRNAs studied, the expression of circulating miR-424-5p was significantly higher, while the expression levels of circulating miR-136-5p were significantly lower in patients with DVT compared with patients without DVT [80]. The authors concluded that the aberrant expression of circulating miR-424-5p and miR-136-5p was associated with hypercoagulability. At the same time, one of the studies demonstrated the action of endothelial progenitor cell-released extracellular vesicles (EPC-EVs) for transferring miR-136-5p in DVT [81]. It was shown that initially, miR-136-5p expression was suppressed and that the thioredoxin-interacting protein (TXNIP) expression was elevated in DVT mice. In the results, the EPC-EV transfer of the miR-136-5p agomir reduced the length and weight of venous thrombi, suppressed cell apoptosis and inflammatory reactions, elevated levels of plasmin, and reduced levels of fibrinogen and thrombin–antithrombin in DVT mice. However, the role of miR-424-5p in the mechanism of DVT has not been fully explored. Previous research has shown that miR-424 regulates important cellular functions, including differentiation, proliferation, and the cell cycle [82]. MiR-424 has been reported to be upregulated following vascular injury and regulates endothelial angiogenic factors by targeting vascular endothelial growth factor receptor-2 (VEGFR-2) and fibroblast growth factor receptor-1 (FGFR-1) [83,84]. The overexpression of miR-424 can reduce EC proliferation, migration, and capillary tube formation. It is known that regeneration and vasculogenesis contribute to the formation and resolution of blood clots. It is possible that changes in the expression level of circulating miR-424-5p and miR-136-5p in the plasma of patients with DVT correspond to the biological functions of miR-424-5p and miR-136-5p.

Qin et al. systematically measured serum concentrations of three circulating miRNAs (miR-582, miR-195, and miR-532) in patients following orthopedic surgery complicated by lower extremity DVT [85]. The expression of these circulating miRNAs was found to be significantly increased in the serum of patients with complicated lower extremity DVT. In addition, receiver operating characteristic (ROC) analysis demonstrated diagnostic significance for these three circulating miRNAs for DVT (area under the curve (AUC): miR-582, 0.959; miR-195, 1.000; miR-532, 1.000). In addition, Xie et al. demonstrated a correlation between circulating miR-96 and plasma D-dimer levels in patients with DVT after orthopedic surgery [86]. Increased circulating miR-96 expression levels correlated with plasma D-dimer levels in postoperative DVT patients. However, although circulating miR-96 expression levels were also increased in the group of patients without DVT after surgery, circulating miR-96 expression levels did not correlate with D-dimer levels in this group. The simultaneous detection of increased levels of circulating miR-96 and D-dimer expression may facilitate more accurate diagnoses of DVT after surgery.

In a study by Jiang et al., high expression levels of plasma miR-320b were found in DVT patients compared with control groups [87]. The ROC curve analysis showed that circulating miR-320b has diagnostic potential with an AUC of 0.79 (sensitivity 69% and specificity 90%) in DVT. In addition, increased circulating miR-320b expression levels in plasma correlated with D-dimer levels.

Previously, it has been proven that the miR-320 family is overexpressed in human platelets, while circulating miR-320b could possibly be transferred through extracellular vesicles (EVs) to ECs with the control of the intercellular adhesion molecule type 1 (ICAM-1) expression, which indicates the potential role of existing extracellular communications in the vascular bed [88].

The identification of circulating miRNAs associated with DVT is of great interest and has the potential to significantly impact the diagnosis and monitoring of therapies for this disease. The use of circulating miRNAs in clinical medicine as diagnostic and prognostic biomarkers is the subject of extensive study but is very far from practical implementation. Table 4 summarizes the results of other studies that have assessed some representative circulating miRNAs as non-invasive biomarkers in DVT [47,48,55,59,85,87,89,90].

### 5.2. In PE

Another critical dimension of PE research revolves around enhancing its diagnostic accuracy. Diagnosing pulmonary embolism remains a formidable challenge due to its nonspecific clinical symptoms and the absence of highly reliable biomarkers. Nevertheless, recent studies have explored the potential utility of circulating miRNAs as diagnostic tools. In one such study, researchers scrutinized the expression levels of circulating miR-190 and miR-197 in patients with PE [91]. These investigators collected plasma samples from individuals with PE, myocardial infarction (MI) patients, and healthy controls. Both circulating miR-190 and miR-197 were found to be significantly elevated in the PE group compared to the MI group and healthy controls. When assessed as diagnostic indicators, these circulating miRNAs demonstrated notable sensitivity and specificity. Circulating miR-190 achieved an AUC of 0.7844, while circulating miR-197 reached an AUC of 0.7931. Furthermore, combining these circulating miRNAs with D-dimer levels significantly improved the diagnostic accuracy, resulting in an impressive AUC of 0.9536. These results underscore the potential of circulating miR-190 and miR-197 as non-invasive diagnostic markers for PE, particularly when utilized in conjunction with D-dimer levels. This synergistic approach holds the promise of significantly enhancing the precision of PE diagnosis, a long-standing challenge in the field.

Xiao et al. showed the involvement and possibility of using circulating miR-134 in the diagnosis and prognosis of PE [92]. The expression level of circulating miR-134 in plasma was increased in the acute PE patient group compared to both the healthy control and non-acute PE patient groups. Furthermore, the authors compared the circulating miR-134 levels between the high-intermediate-risk acute PE and low-risk acute PE groups and found that the expression level of circulating miR-134 was significantly higher in the high-intermediate-risk acute PE patients compared to the low-risk patients. In addition it was demonstrated that the circulating miR-134 level was not affected by non-acute PE conditions. Circulating miR-134 could distinguish acute PE cases from healthy controls or non-acute PE cases, with an AUC of 0.833 or 0.756, respectively, which indicates the potential for using circulating miR-134 as a diagnostic biomarker.

Wang et al. examined the levels of circulating miR-27a and miR-27b as diagnostic biomarkers in patients with PE [93]. The results showed that patients with acute PE had increased levels of circulating miR-27a and miR-27b relative to the control group. The ROC curve analysis showed that circulating miR-27a was superior to miR-27b for the diagnosis of acute PE, with AUCs of 0.784 and 0.707, respectively. The simultaneous measurement of D-dimer levels and circulating miR-27a/miR-27b levels significantly increased the diagnostic sensitivity in establishing the diagnosis of PE.

Lastly, the diagnosis of the acute exacerbation of chronic obstructive pulmonary disease (COPD) complicated by PE presents unique diagnostic challenges due to the variability in clinical presentations and the absence of reliable screening biomarkers or instruments [94]. To address this diagnostic conundrum, researchers examined serum miRNAs, specifically miR-1233 and miR-134, in COPD patients with and without PE [95]. This study encompassed 52 COPD patients, with 13 of them diagnosed with PE alongside 39 COPD patients without PE and 10 stable COPD patients. The results revealed that circulating miR-1233 and miR-134 levels were significantly elevated in COPD patients with PE compared to those without PE and stable COPD patients. Notably, these circulating miRNAs outperformed conventional measures, such as D-dimer levels and the Wells score, in terms of diagnostic accuracy. In conclusion, circulating miR-1233 and miR-134 demonstrate substantial clinical promise in the early diagnosis of COPD patients with or without PE complications. These miRNAs may serve as invaluable biomarkers for distinguishing between these complex clinical conditions in real-world medical practice. Two specific circulating miRNAs, namely, miR-145 and miR-126, have emerged as potential biomarkers for distinguishing COPD patients with and without PE [96]. The results of these studies have shown that circulating miR-145 levels tend to be lower in COPD patients with PE compared to those without PE, while circulating miR-126 levels are elevated in COPD patients with PE. Notably, circulating miR-145 exhibited positive correlations with lung function (the forced expiratory volume in 1 s (FEV1)/forced vital capacity (FVC) ratio) and negative correlations with D-dimer levels, a marker of clot formation, in all the patients, regardless of the presence of PE. Conversely, circulating miR-126 demonstrated positive correlations with D-dimer levels and negative correlations with lung function in all the COPD patients. These intriguing findings suggest that lower circulating miR-145 levels and higher circulating miR-126 levels may be associated with worse prognoses in COPD patients who develop PE. However, it is imperative to underscore that further comprehensive research is essential to fully elucidate the precise roles of these miRNAs in thrombosis within the complex context of COPD.

One intriguing area of exploration lies in the intersection between PE and CTEPH. It is known that CTEPH is the most severe complication of PE, making it a focal point for research. The tasks of early diagnosis for patients with CTEPH are aimed at the timely establishment of the operability of patients because a successful pulmonary thromboendarterectomy leads to the regression of pulmonary hypertension in most patients [97].

Guo et al. analyzed plasma samples from patients with CTEPH and healthy donors and found that circulating let-7b expression was positively correlated with the PAI-1 levels, D-dimer, and cardiac index [98]. In addition, the ROC curve analysis revealed that circulating let-7b had a moderate value for CTEPH diagnosis, with an area under the curve of 0.769 (95% CI: 0.664–0.874). Based on the results of in silico studies, the main targets of this microRNA were found—endothelin receptor-1 and transforming growth factor-β (TGF-β). It was observed that a decrease in circulating let-7b expression in PASMCs correlates with an increase in endothelin-1 levels. In another study, the same authors demonstrated decreased expressions of circulating let-7, miR-17-5p, miR-106b-5p, and miR-93-5p in patients with CTEPH relative to controls [99]. At the same time, the expression levels of circulating miR-3202 and miR-665 were significantly higher. In addition, a positive correlation was observed between the miR-93-5p expression and N-terminal pro B-type natriuretic peptide (NT-proBNP) level, while a negative correlation was observed between the miR-93-5p expression and CI (r = −0.861, *p* < 0.05). The let-7b-3p expression showed a trend toward a positive correlation with the mean pulmonary arterial pressure (mPAP).

In summary, these studies collectively highlight the burgeoning significance of circulating miRNAs in the realm of VTC research. From miRNAs’ pivotal roles in comprehending the pathophysiology of this condition to their potential as predictive, diagnostic, and prognostic non-invasive biomarkers, circulating miRNAs represent a beacon of hope for the enhanced identification of patients with problems and more effective management of patients with DVT and PE and their related conditions. As we continue to unravel the complexities of VTC processes, miRNAs offer the potential for transformative breakthroughs in this critical area of medicine. Table 5 summarizes the results of other studies that assessed some representative circulating miRNAs as non-invasive biomarkers in PE [100,101,102,103].

## 6. Perspectives and Limitations

Close attention to miRNA-based therapy for various human diseases, as a promising tool for targeting gene expression, is explained by the following factors: (1) specificity of action, (2) minor side effects, and (3) ease of synthesis of miRNA-based drugs. Correctly selected RNA sequences can selectively inhibit the expression of genes associated with certain disorders, such as the overexpression of genes responsible for blood clots. Currently, according to information from ClinicalTrials.gov, more than 30 miRNA-based drugs are in Phase 3–4 clinical trials. Despite the great potential for using miRNA for therapeutic purposes, only a few drugs have had satisfactory results. Under physiological conditions, miRNA is unstable. The effective action of miRNAs involves their delivery to target cells through the bloodstream; however, when administered intravenously, the molecules are degraded under the action of serum nucleases, excreted by the kidneys, absorbed by phagocytes, and aggregated with whey proteins. Nuclease activity in blood serum and tissues is, in fact, the first barrier to miRNA delivery. The half-life of siRNA in the bloodstream ranges from several minutes to 1 h. The delivery vehicles that are used today, including nanoparticles and liposomes described in many studies, are characterized by high toxicity, poor selectivity, and low efficiency in penetrating cells. It is because of this that miRNA preparations for external use are being actively developed, mainly for the treatment of eye diseases and skin diseases, in which the issues of specific delivery are partially removed. Regarding the treatment of DVT and PE, there are currently no active clinical trials. Despite the specificity of action of miRNAs and their ability to selectively block the activity of individual protein genes, such drugs cannot be expected to be effective in the treatment of cancer, even if they successfully block the expression of key proteins. One of the reasons for this may be that thrombosis is a complication of the underlying disease, such as cancer. Therefore, therapies may be complicated by the choice of tactics. One approach to increasing the effectiveness of targeted compounds is the use of a combination of drugs with multidirectional effects that simultaneously block several signaling pathways in the cell/cells.

Although circulating-miRNA expression analysis has been studied worldwide, this research has mainly focused on applications in the fields of biomedicine and oncology. To truly unlock the full potential of circulating miRNAs in DVT and PE, it is critical to understand the molecular complexity of the available samples and the hemostatic system itself. There are not very many published studies in this area, but their number is growing. The success of their implementation in clinical practice will largely depend on the availability of a method that allows for the effective verification and validation of promising biomarkers based on circulating miRNAs. Frequent inconsistencies, and sometimes contradictions, in research results are associated with the difficulty in determining the functional involvement of a specific miRNA in the development of a particular process owing to the simultaneous effects of the miRNA on several target genes. As mentioned above, thrombosis is often a complication of the underlying disease. In other words, the use of circulating miRNAs for diagnostic purposes will be limited until their precise relationships with genes are established.

## 7. Conclusions

Research over the past few years has shown that miRNAs serve as modulators of hemostasis. Understanding the mechanism by which microRNAs are involved in the development of thrombosis is especially important because it can be applied in clinical practice. Circulating miRNAs have enormous potential as non-invasive biomarkers for use in the diagnosis and prognosis of VTCs. However, there are several limitations, such as the lack of studies examining both endogenous and circulating miRNAs in DVT and PE. Another limitation stems from the fact that thrombosis is a dynamic process that is influenced by multiple factors, such as primary diseases, the timing of sampling, and prophylactic anticoagulation. Consequently, there is clinically significant heterogeneity among the study population. Inevitably, problems will arise because one miRNA can have hundreds of target genes, and one gene can be the target of many miRNAs. The formation of a blood clot in the venous system is a rather complex process controlled by many miRNAs. It is very important, but extremely difficult, to determine the exact mechanisms that are regulated by specific miRNAs, so further research is needed to solve this problem.

## Figures and Tables

**Table 1:** The role of microRNAs (miRNAs) in thrombosis.

Thrombosis/Conditions Associated with Thrombosis	miRNA	Regulation	Study	Targets	Effect	References
AAF	miR-200b-3p	Up	In vitro	VEGF and Ang-II	Increases intimal hyperplasia to induce autogenous arteriovenous fistula thrombosis	[7]
Thrombosis	miR-1915-3p	Up	In vitro	RHOB	Enhances megakaryocyte (megakaryopoiesis) differentiation and platelet generation	[8]
COVID-19	miR-145 and miR-885	Down	Ex vivo (human serum) and in vitro	D-dimer	Increase endothelial cell apoptosis and display significantly impaired angiogenetic properties	[9]
Inflammation and arterial thrombosis	miR-146b-3p	Up	In vitro	P38MAPK/COX-2 pathway	TNF-α activates miR-146b-3p and then induces thrombosis	[10]
COVID-19	miR-16-5p	Down	In silico and bioinformatic analysis	EGFR, HSP90AA1, APP, TP53, PTEN, UBC, FN1, ELAVL1, and CALM1	Thrombosis-related predictive biomarker	[11]
Multiple myeloma	miR-532	Down	Ex vivo (human serum) and in vitro	TF, EPCR, ERK 1/2, MAPK, and NF-κB	Increases multiple myeloma-induced endothelial cell thrombosis	[12]
Photochemical-induced carotid thrombosis	miR-223	Down	In vivo	IGF-1R	Attenuates thrombosis	[13]
Primary antiphospholipid syndrome	miR-483-3p miR-326	Up Down	In vitro	PI3K/AKT and NOTCH	Contribute to primary antiphospholipid syndrome pathogenesis by producing endothelial cell proliferation, monocyte activation, and adhesion/procoagulant factors	[14]
Atherosclerotic plaque rupture with thrombosis	miR-466h-5p	Up	In vivo	Bcl2	Promotes atherosclerotic plaque rupture and thrombosis	[15]
Catheter-related thrombosis	miR-92a-3p	Down	In vivo	MAPK/NF-κB	Inhibits oxidative stress injury and prevents catheter-related thrombosis formation	[16]

Abbreviations: AAF, Autogenous arteriovenous fistula; COVID-19, Coronavirus disease 2019; VEGF, Vascular endothelial growth factor; Ang-II, Angiotensin II; RHOB, Rho GTPase family member B; P38MAPK, p38 mitogen-activated protein kinases; COX-2, Cyclooxygenase-2; EGFR, epidermal growth factor receptor; HSP90AA1, Heat shock protein 90 alpha family class A member 1; APP, Amyloid-beta precursor protein; PTEN, Phosphatase and tensin homolog; UBC, Ubiquitin C; FN1, Fibronectin 1; ELAVL1, ELAV-like protein 1; CALM1, Calmodulin 1; TF, Tissue factor; EPCR, Endothelial protein C receptor; ERK 1/2, Extracellular signal-regulated kinase; MAPK, p38 mitogen activated protein kinase; NF-κB, Nuclear factor-κB; TNF-α, Tumor necrosis factor alpha; IGF-1R, insulin-like growth factor 1; PI3K, Phosphoinositide 3-kinases; NOTCH, Neurogenic locus notch homolog protein 1; Bcl2, B-cell lymphoma 2.

**Table 2:** Shows the list of some representative miRNAs that are involved in the process of deep vein thrombosis (DVT).

miRNA	Regulation	Study Model	Targets	Biological Effect	References
miR-206	Down	In vitro (human EPCs)	GJA1	Inhibits autophagy of EPCs and promotes EPC proliferation, migration, and angiogenesis, thereby enhancing EPC homing to thrombi and facilitating thrombus resolution	[46]
miR-374b-5p	Up	In vitro (293T cells and HUVECs) and in vivo (C57BL/6J mice)	IL-10	Promotes DVT formation	[47]
miR-296-5p	Up	In vitro (HUVECs) and in vivo (C57BL/6J mice)	S100A4	Inhibits DVT formation	[48]
miR-5189-3p	Up	In vivo (SPF-grade SD rats)	JAG1, Notch1, and Hes1	Inhibits apoptosis and significantly increases the recanalization of venous thrombosis	[49]
miR-181a-5p	Up	In vivo (C57BL/6J mice)	IL-1β, IL-6, and IL-8	Attenuates vascular inflammatory injury to curtail DVT	[50]
miR-483-3p	Up	In vitro (human EPCs) and in vivo (male SD rats)	SRF	Decreases EPC migration, tube formation, homing, and thrombus resolution and increases apoptosis	[51]
miR-125a-3p	Up	In vivo (male SD rats)	IL1R1, IL-6, and IL-8	Represses inflammation and thrombosis	[52]
miR-342-3p	Up	In vitro (HUVECs) and in vivo (male SD rats)	EDNRA	Inhibits DVT formation and improves angiogenesis	[53]
miR-195-5p	Down	In vitro (HUVECs)	Bcl-2	Promotes cell viability and inhibits apoptosis; involved in the development of DVT	[54]
miR-181c-5p	Up	In vitro (HUVECs)	FOS	Protective effect; involved in DVT progression by mediating EC injury	[55]
miR-125a-5p	Down	In vitro (human EPCs) and in vivo (C57BL/6J mice)	MCL-1 and PI3K/AKT pathways	Promotes EPC migration and angiogenesis, thereby enhancing EPC homing to thrombi and facilitating thrombus resolution	[56]
let-7e-5p	Up	In vitro (human EPCs) and in vivo (male SD rats)	FASLG	Improves EPC homing and thrombus revascularization	[57]
miR-103a-3p	Up	In vitro (human EPCs)	PTEN	Promotes EPC migration and angiogenesis, thereby enhancing EPC homing to thrombi and facilitating thrombus resolution	[58]
miR-128-3p	Up	In vitro (HUVECs)	SIRT1	Suppresses cell proliferation, inhibits cell migration, and promotes cell death; promotes DVT formation	[59]
miR-143-3p	Up	In vitro (human EPCs)	ATG2B	Enhances viability, migration, invasion, and tube formation of EPCs; inhibits DVT formation	[60]
miR-205	Up	In vitro (human EPCs) and in vivo (male nude mice)	PTEN and MMP2	Promotes EPC homing to the thrombus, and DVT recanalization and resolution; promotes EPC angiogenesis, increases cell proliferation, and inhibits apoptosis	[61]

Abbreviations: EPCs, Endothelial progenitor cells; HUVECs, Human umbilical vein endothelial cells; SD rats, Sprague–Dawley rats; GJA1, Gap junction protein alpha 1; IL-10, Interleukin 10; S100A4, S100 Calcium Binding Protein A4; JAG1, Jagged1; Notch1, Neurogenic locus notch homolog protein 1; Hes1, Hairy and enhancer of split-1; IL-1β, Interleukin 1β; IL-6, Interleukin 6; IL-8, Interleukin 8; SRF, Serum response factor; IL1R1, Interleukin 1 receptor, type I; EDNRA, Endothelin A receptor; Bcl-2, B-cell lymphoma 2; FOS, FBJ osteosarcoma oncogene; MCL-1, Myeloid leukemia 1; PI3K, Phosphoinositide 3-kinases; FASLG, Fas ligand; PTEN, Phosphatase and tensin homolog; SIRT1, Silent information regulator sirtuin 1; ATG2B, Autophagy-related 2B; MMP2, Matrix metalloproteinase-2.

**Table 3:** Shows the list of some representative miRNAs that are involved in the process of pulmonary embolism (PE).

miRNA	Regulation	Study Model	Targets	Biological Effect	References
Exosomal miR-28-3p	Up	In vitro (human PECs and mouse PECs) and in vivo (C57BL/6J mice)	API5	Promotes PECs apoptosis	[68]
miR-514a-5p	Up	In vitro (human PASMCs) and in vivo (male SD rats)	CHRDL1	Promotes the progression of PE by promoting inflammation and lung injury	[69]
miR-106b-5p	Up	In vitro (human PASMCs) and in vivo (C57BL/6J mice)	NOR1	Improves acute PE-induced mortality and pulmonary vascular proliferation	[70]
miR-21	Up	In vivo (SD rats) and ex-vivo (rats’ whole blood)	PTEN/NF-κB axis	Enhances lung injury and inflammation in acute PE; increases PASP and RVSP levels, W/D ratio, and thrombus volume	[71]
let-7b-5p	Up	In vivo (specific pathogen-free rabbits) and in vitro (human lung epithelial cells (BEAS-2B)	SERP1	Involved in ER stress response, promotion of cell apoptosis, and induction of lesions and inflammation in lung tissues	[72]
let-7b-5p	Up	In vitro (human PASMCs)	PDGF/IGF-1 axis	Suppresses proliferation and migration of PASMCs	[73]

Abbreviations: PEC, Human pulmonary endothelial cells; PASMCs, Human pulmonary artery smooth muscle cells; SD rats, Sprague–Dawley rats; API5, Apoptosis inhibitor 5; CHRDL1, Chordin-like 1; NOR1, Nuclear receptor 4A3; PTEN, Phosphatase and tensin homolog; NF-κB, Nuclear factor kappa-light-chain-enhancer of activated B cells; SERP1, Stress-associated endoplasmic reticulum protein 1; PDGF, Platelet-derived growth factor; IGF-1, Insulin-like growth factor 1; PASP, Pulmonary systolic artery pressure; RVSP, RV systolic pressure.

**Table 4:** Circulating microRNAs (miRNAs) as biomarkers in deep vein thrombosis (DVT) patients.

miRNA	Number of Patients/Control Group, *n*	Regulation	Biofluid	AUC	Sensitivity, %	Specificity, %	Important Findings	References
miR-374b-5p	36/36	Up	Venous blood (PBMCs)	0.834	-	-	Independent and diagnostic biomarker and powerful predictor	[47]
miR-296-5p	41/41	Up	Venous blood (Plasma)	0.88	-	-	Independent and diagnostic biomarker and powerful predictor	[48]
miR-130a-3p, miR-181c-5p, miR-196b-5p, and miR-4467	45/45	Down	Peripheral blood	0.844, 0.880, 0.757, and 0.785	-	-	Independent and diagnostic biomarkers and powerful predictors	[55]
miR-128-3p	79/79	Up	Peripheral blood	0.920	0.888	0.831	Independent and diagnostic biomarker and powerful predictor	[59]
miR-582, miR-195, and miR-532	18/20	Up	Venous blood (Serum)	0.959, 1.000, and 1.000	-	-	Independent and diagnostic biomarkers and powerful predictors	[85]
miR-320b	30/30	Up	Venous blood (Plasma)	0.79	-	-	Independent and diagnostic biomarker and powerful predictor; correlates to D-dimer levels	[87]
miR-125a-5p and miR-223-3p	30/30	Up and down	Venous blood (Plasma)	>0.8	>0.97	>95	Most significantly changed in patients with DVT before and after endovascular interventions; independent and diagnostic biomarkers and powerful predictors; correlate to D-dimer levels	[89]
miR-448	40/72	Up	Venous blood (PBMCs)	-	-	-	Independent risk factor for postoperative DVT; MiR-488 combined with SIRT1 has a high predictive value for occurrence of DVT.	[90]

Abbreviations: PBMCs, Human peripheral blood mononuclear cells; AUC, area under curve; ROC, receiver operating characteristic; AUC is considered predictive and diagnostically significant for the biomarker, ≥0.75; -, not mentioned in the article.

**Table 5:** Circulating microRNAs (miRNAs) as biomarkers in pulmonary embolism (PE) and its complications.

Disease	miRNA	Number of Patients/Control Group, *n*	Regulation	Biofluid	AUC	Sensitivity, %	Specificity, %	Important Findings	References
APTE, CTEPH, and IPAH	let-7i-5p and miR-320a	19, 14, and 14/13	Up	Plasma	-	-	-	Circulating miRNAs in baseline and clinical characteristics of patients reinforce differences between APTE and CTEPH in outcome evolution, as well as differences between CTEPH and IPAH in disease form	[100]
PE and acute NSTEMI	miR-1233	30 and 30/12	Up	Serum	0.95 and 0.91	90.0 and 90.0	100.0 and 92.0	Distinguishing acute PE from acute NSTEMI and healthy individuals with high specificity and sensitivity	[101]
Acute PE	miR-221	60/50	Up	Plasma	0.823	-	-	Potential diagnostic biomarker; positively correlates with plasma concentrations of BNP, troponin I, and D-dimer	[102]
PE	miR-28-3p	37/37	Up	Plasma	0.792	-	-	Potential diagnostic biomarker	[103]

Abbreviations: APTE, Acute pulmonary embolism; CTEPH, Chronic thromboembolic pulmonary hypertension; IPAH, Idiopathic pulmonary artery hypertension; NSTEMI, Non-ST-elevation myocardial infarction; BNP, Brain natriuretic peptide; AUC, area under curve; ROC, receiver operating characteristic; AUC is considered predictive and diagnostically significant for the biomarker, ≥0.75; -, not mentioned in the article.
